# Supplementary material for: Collaborative intelligence in AI: Evaluating the performance of a council of AIs on the USMLE
Source: PLOS Digit Health. 2025 Oct 9;4(10):e0000787. doi: 10.1371/journal.pdig.0000787 (PMC12510544; doi:10.1371/journal.pdig.0000787)
Supplement: S2 File — (ZIP) [file pdig.0000787.s002.zip › S2 Data Raw data files containing unprocessed question inputs and LLM outputs including transcripts of deliberations/STEP3_Question-108-1_Type-MC-NJ.docx]

**Facilitator** **[Round 1]:** A 5-year-old boy returns to the office with his mother to discuss results of studies done to evaluate his behavior of eating dirt. The patient sits quietly while his mother answers questions about his health. She says her son seems healthy, although he does not seem to be as active as other children his age. He has said his head hurts three or four times during the past 2 weeks. He has not had fever, sweats, or chills. She says he has a good appetite but has had a habit of eating sand and dirt since age 3 years. She says, ""I don't know where he gets that habit. I used to eat dirt, but I stopped years ago. I try to stop him from eating dirt, but I'm not around much since I work two jobs."" The patient takes no medications. Vaccinations are up-to-date. Height, weight, and BMI are at the 50th percentile. Vital signs are normal. Physical examination discloses no abnormalities except for symmetrical nontender cervical adenopathy. Results of laboratory studies, including serum zinc, lead, and iron concentrations, are within the reference ranges. Serologic testing confirms toxoplasmosis. In addition to prescribing pyrimethamine and sulfadiazine therapy, which of the following is the most appropriate next step in management? (A) Prescribe fluoxetine (B) Prescribe methylphenidate (C) Prescribe risperidone (D) Refer the patient to a child psychiatrist (E) Refer the patient to a dietician

**AI_D:** RESTATING THE QUESTION: This is about a 5-year-old boy who has a history of regularly consuming dirt, also known as pica. His mother states that he exhibits less activity than his peers and occasionally complains of headache. He has symmetrical cervical adenopathy, and his blood tests indicate the presence of Toxoplasma gondii parasitic infection but otherwise normal nutritional parameters. He is currently being treated with antiparasitic drugs pyrimethamine and sulfadiazine. We need to decide what the next step in managing his health is, choosing between prescribing an antidepressant (fluoxetine), a stimulant (methylphenidate), an antipsychotic (risperidone), referring to a child psychiatrist, or referring to a dietician. PLANNING: There are several scenarios that could explain this child’s behavior and symptoms. First, pica could be the cause, which is an eating disorder that involves consuming nonfood items. Additionally, pica is often associated with micronutrient deficiencies, although in this case blood tests don't show it. Toxoplasma infection could also cause his symptoms, as it can manifest with lymphadenopathy and systemic signs. Additionally, psychiatric illnesses can often manifest with physical symptoms like headache. Each of these hypotheses needs to be evaluated. CLINICAL REASONING: Given that his serum levels of key micronutrients involved in pica (iron, zinc, lead) are within normal limits, it is less likely that nutritional deficiencies are driving his pica. Besides, the successful treatment of toxoplasmosis may not change his pica behavior since it started before the infection. Since mental health issues can cause behavioral changes and physical discomfort symptoms like headache, it may be prudent to assess the child’s mental and emotional health. Therefore, a referral to a child psychiatrist may be warranted for further evaluation. CONCLUDING: In assessing a child with behavior like pica, it's a multidimensional assessment process where we consider the physical, nutritional, as well as the mental health of the child. In this scenario, considering the child's behavior, unspecified headache, and absence of nutritional-deficiency, a further psychiatric evaluation may provide a more comprehensive understanding of the underlying cause. RESPONSE: Therefore, I believe that the most appropriate next step in managing this child's health is to refer the patient to a child psychiatrist (option D).

**AI_E:** RESTATING THE QUESTION: The question describes a 5-year-old boy who displays certain striking behavioural features, namely eating dirt (a condition known as pica), as well as less pronounced activities compared to his peers. The child is overall healthy and has normal weight with no other abnormalities apart from symmetrical nontender cervical adenopathy. Recent serological tests have confirmed toxoplasmosis, a foodborne-disease which is often attributed to the consumption of undercooked meat and exposure to cat feces, but could potentially happen due to soil or dirt contamination. The question is asking what the next course of action should be beyond prescribing medicine for his toxoplasmosis. PLANNING: The assumptions here are that the child has a dietary disorder causing pica, a condition characterized by the consumption of items with no nutritional value, and that this is tied to nutritional deficiencies. An alternate assumption could be that dietary preferences are learned behaviors and the child picked up dirt-eating from the mother. It is also important to consider the role of the confirmed toxoplasmosis and its potential for causing decreased activity and headaches in the child. CLINICAL REASONING: The fact that the lab results have come back with normal serum zinc, lead, and iron concentrations is indicative that the issue might not be related to nutrient deficiency, as these are some of the common deficiencies implicated in cases of pica. Here, other environmental factors and habits must be taken into account, such as the mother's admission of her previous dirt-eating habits. However, it's important to not overlook the presence of toxoplasmosis, as this might be the cause for some of his symptoms. This infection is usually asymptomatic, but it can cause symptoms that are somewhat reminiscent of the flu and could potentially explain the child's intermittent headaches and decreased activity level. This would have been contracted through the ingestion of oocytes that are present in infected soil or cat feces - something that would have arisen from his dirt-eating habit. CONCLUDING: In consideration of the overall situation, the child's previously confirmed toxoplasmosis will likely be resolved with the suggested treatment of pyrimethamine and sulfadiazine. However, in order to treat the pica condition and prevent further risk of infections, it might be beneficial to focus on the behavioral component of the child's dirt-eating habit. RESPONSE: Hence, the next step should be to (D) Refer the patient to a child psychiatrist. This suggestion aligns with the need to address the child's dirt-eating habit which seems to be a learned behaviour rather than due to a physiological need. A child psychiatrist might be able to work with the child and possibly the mother to change this concerning behaviour and prevent any future complications.

**AI_A:** RESTATING THE QUESTION: The main issue revolves around a 5-year-old boy who has a habit of eating dirt, and as a result, has been diagnosed with toxoplasmosis. Despite this behavior and confirmed infection, there are no major physical abnormalities and his growth metrics and laboratory tests are within normal ranges. Toxoplasmosis, a parasitic disease often transmitted via ingestion of contaminated soil, has been confirmed. The mother tries to prevent his dirt-eating behavior, but sometimes finds it challenging due to her work schedule. The question now is, what should be done aside from treating his toxoplasmosis with pyrimethamine and sulfadiazine? PLANNING: The underlying assumption in this case is that his dirt-eating habit (pica), while resulting in toxoplasmosis, doesn't appear to be impacting his general state of health too significantly - at least from what is immediately observable. One alternative assumption could be that this behavior might still have other psychological, emotional or nutritional implications that needs to be addressed. Understanding the reason behind his pica could be a key aspect of the overall management. CLINICAL REASONING: The child's pica can be a sign of nutritional deficiency, but his tests - including those for zinc, lead, and iron - are within the reference ranges, suggesting no immediate, severe deficiency. However, the persistent pica behavior coupled with maternal history of the same phenomenon might suggest a psychological component that could be addressed by professionals in child psychology or nutrition, who can understand the motivations behind the child's behavior and how to manage it. CONCLUDING: While the planned treatment for toxoplasmosis is appropriate, addressing the root cause i.e., the child's behavior of eating dirt, is vital to prevent future health risks. Although there doesn't appear to be a biochemical abnormality needing supportive supplementation (e.g., iron), the persistent nature of the child's behavior suggests that other intervention, such as consultation with a child psychiatrist or a dietician could be beneficial. RESPONSE: Therefore, the most appropriate next step in management, along with the prescription of pyrimethamine and sulfadiazine, is to refer the patient to a dietician (E). The dietician can provide recommendations for nutritional changes if necessary or strategies to help modify the child's eating behavior. A referral to a child psychiatrist (D) could also be considered as a secondary measure if pica persists or if there are other behavioral or emotional concerns. The prescription of medications like fluoxetine (A), methylphenidate (B), or risperidone (C) doesn't seem directly related to the child's clinical presentation and may not be the most beneficial first line management at this stage.

**AI_C:** 1) RESTATING THE QUESTION: The presented case talks about a 5-year-old boy who has been eating dirt since he was 3 years old. He has been relatively inactive for his age and has recently complained of headaches. No other symptoms like fever, sweats, or chills have been reported. He also shows enlarged lymph nodes in the neck region but has normal vital signs and his growth measures fall within the 50th percentile. All the laboratory tests including measurements for serum zinc, lead, and iron, are within the normal reference range, but the serologic testing has confirmed toxoplasmosis, a disease caused by an intracellular protozoan, Toxoplasma gondii. It is often associated with consumption of uncooked meat, exposure to infected cat feces, or contaminated soil, which can fit in with the habit of eating dirt as is seen in the boy's case. The patient's treatment plan includes pyrimethamine and sulfadiazine, but there is a query as to what constitutes the most appropriate next step in managing his case. 2) PLANNING: The assumption based on the current case could be that the boy's abnormal behavior and toxoplasmosis are connected. Yet, an alternate assumption might indicate they are two separate conditions - the boy's consumption of dirt being a behavioral manifestation potentially related to his home setting and toxoplasmosis coming from the nature of what he consumes. On evaluating these assumptions, the second seems to be more likely because the mother has admitted to a similar habit in the past and her inability to keep a check on her son due to her work schedule. 3) CLINICAL REASONING: As per the information, the boy's physical growth and development are normal as height, weight, and BMI are at the 50th percentile. The only physical abnormality found was the symmetrical nontender cervical adenopathy, indicative of an infection, which was later confirmed by tests as toxoplasmosis. Pyrimethamine and sulfadiazine are usually effective against toxoplasmosis and should be continued. Symptoms such as relative inactivity and headaches, coupled with an unusual habit of consuming dirt, suggest a potential behavioral or developmental issue that could be better addressed by a child psychiatrist. The medications listed as options (fluoxetine, methylphenidate, risperidone) are usually used in psychiatric conditions such as depression, ADHD, and schizophrenia and should not be prescribed without a clear diagnosis made by a mental health professional. 4) CONCLUDING: Taken together, with underlying toxoplasmosis being managed with targeted pharmacologic intervention (pyrimethamine and sulfadiazine), the boy's unusual behavior of ingesting dirt (not related to a nutritional deficiency) and the mother's history of similar behavior emphasizes the need for psychiatric assessment. This could potentially uncover any underlying behavioral or mental health issues that might be leading to his abnormal eating habit. 5) RESPONSE: As such, the most appropriate next step in this young patient's management would be to 'Refer the patient to a child psychiatrist' (D).

**AI_B:** This clinical case presents a 5-year-old boy whose mother has observed him consuming sand and dirt. Despite this unusual habit, the child's physiological measurements and vital signs appear to be standard for his age group. More importantly, laboratory tests, which included serum zinc, lead, and iron levels, showed no abnormalities. His examination however revealed symmetrical, nontender cervical adenopathy, and he tested positive for toxoplasmosis serologically. The dilemma we now face is determining the next best step in his care post-operationalizing the treatment for toxoplasmosis. The options presented for this course of action are prescribing fluoxetine, methylphenidate, risperidone, referring the patient to a child psychiatrist, or referring the patient to a dietician. Ideally, our choice has to address the primary behavior anomaly observed in this child, which is the dirt-eating habit, clinically known as pica—an unusual craving for non-nutritive substances such as clay, paint, or dirt. This condition commonly manifests in children, pregnant women, and individuals with developmental disabilities. In the absence of any presently visible mental disorder, the options for prescribing mental health medication may not be primarily needed. Fluoxetine is for the management of major depressive disorder, obsessive-compulsive disorder, panic disorder, and social phobia; methylphenidate is used to handle attention deficit hyperactivity disorder and narcolepsy; and risperidone is for the treatment of schizophrenia symptoms, bipolar disorder, and irritability linked with autistic disorder. Consequently, our assumption leans primarily towards the behavioral and dietary factors that may contribute to the manifestation of this condition. Given the mother's absence at home due to work, it could suggest lack of proper dietary supervision. As such, the alternate assumption in coordinating the management treatment plan for this patient should prioritize the need to address his unusual consumption habit. Having delineated the potential reasons for the boy's pica, we may propose that a dietician's intervention might help in assessing and improving the child's nutritional status. The importance of this approach is underscored by the fact that toxoplasmosis, an infection that the boy has contracted, can potentially be linked to his consumption of contaminated soil or sand, given the nature of the parasite's lifecycle. Proceeding from this line of reasoning, managing pica would require establishing a comprehensive nutritional plan that ensures the child gets all the required nutrients to complement the therapeutic approach for toxoplasmosis. Plus, a child's dietary habits possibly evolving from the mother's past behaviors might suggest inherited dietary hurdles a dietician's counsel can address elements such as replacing non-nutritive substances with appropriate meals, diverting attention towards safer activities, and strengthening the nutritional supplement. In conclusion, given the boy's confirmed normal serum zinc, lead, and iron levels, further supportive therapeutic intervention outside his toxoplasmosis treatment would involve addressing the eating habit. In this context, a dietician's expertise can guide the child and his mother in developing healthier nutritional habits to possibly curb the craving for dirt, minimize the risk of further parasitic infection, and improve the boy's overall health status. Therefore, according to my clinical perspective of the case, the most appropriate next step for this boy's management is E: Refer the patient to a dietician.

**Facilitator** **[Round 2]:** Summary: AI_A suggests that the child's pica behavior might have psychological, emotional, or nutritional implications that need to be addressed. They recommend referring the child to a dietician and possibly a child psychiatrist if the pica persists or if there are other behavioral or emotional concerns. AI_B believes that the child's pica is primarily due to behavioral and dietary factors. They recommend referring the child to a dietician to assess and improve the child's nutritional status and to help develop healthier nutritional habits. AI_C thinks that the child's pica and toxoplasmosis are two separate conditions. They suggest that the child's consumption of dirt is a behavioral issue potentially related to his home setting. They recommend referring the child to a child psychiatrist for further evaluation. AI_D believes that the child's pica is not due to nutritional deficiencies but could be due to mental health issues. They recommend referring the child to a child psychiatrist for further evaluation. AI_E suggests that the child's pica is a learned behavior rather than due to a physiological need. They recommend referring the child to a child psychiatrist to work with the child and possibly the mother to change this behavior. Question to clarify disagreements: The main disagreement among the council members is whether the child should be referred to a dietician or a child psychiatrist. Could the council members elaborate on why they believe their recommended professional (dietician or psychiatrist) would be more effective in addressing the child's pica behavior?. Based on your preceding response, how would you answer the original question: [A 5-year-old boy returns to the office with his mother to discuss results of studies done to evaluate his behavior of eating dirt. The patient sits quietly while his mother answers questions about his health. She says her son seems healthy, although he does not seem to be as active as other children his age. He has said his head hurts three or four times during the past 2 weeks. He has not had fever, sweats, or chills. She says he has a good appetite but has had a habit of eating sand and dirt since age 3 years. She says, ""I don't know where he gets that habit. I used to eat dirt, but I stopped years ago. I try to stop him from eating dirt, but I'm not around much since I work two jobs."" The patient takes no medications. Vaccinations are up-to-date. Height, weight, and BMI are at the 50th percentile. Vital signs are normal. Physical examination discloses no abnormalities except for symmetrical nontender cervical adenopathy. Results of laboratory studies, including serum zinc, lead, and iron concentrations, are within the reference ranges. Serologic testing confirms toxoplasmosis. In addition to prescribing pyrimethamine and sulfadiazine therapy, which of the following is the most appropriate next step in management? (A) Prescribe fluoxetine (B) Prescribe methylphenidate (C) Prescribe risperidone (D) Refer the patient to a child psychiatrist (E) Refer the patient to a dietician]

**AI_A:** Starting with our clinical query, we are asked to discern the most appropriate next step in management for a 5-year-old boy who exhibits pica, eating dirt (geophagy), and who has been diagnosed with toxoplasmosis. Pica is the habitual eating of nonfood items, which, in this case, is dirt (geophagy). This child's mother has the same history, indicating that the behavior might be learned. Furthermore, our clinical findings suggest no nutritional deficiency, as his serum zinc, lead, and iron concentrations are within the reference ranges. The presence of toxoplasmosis in this context might be linked to his geophagy. Drawing from these facts, we still need to identify why the habit persists in this child and take appropriate corrective steps. The commonly proposed potential causes are micronutrient deficiencies and underlying psychiatric conditions. However, our case doesn't affirm any micronutrient deficiency, making dietary interventions less likely to correct the behavior. Still, the other potential cause, an underlying psychiatric condition, hasn't been evaluated, making it an alternate assumption we should explore clinically. To further support our hypothesis that psychiatric input might help this child, consider how he has been largely unsupervised due to his mother's work schedule. This lack of supervision could result in a child using self-soothing behaviors, such as pica. Psychiatric input would help address any behavioral or environmental factors contributing to pica, such as stress, neglect, or limited parental supervision. To conclude, although additional nutritional input wouldn't harm the child, a referral to a child psychiatrist could address the root cause of his pica more effectively. Until we can confirm that the cause of his pica is purely nutritional, a psychiatric referral is the most appropriate option. Hence, my final response aligning to the clinical thought process outlined would be to (D) Refer the patient to a child psychiatrist. This response prioritizes the need for a more comprehensive understanding of the child's habitual dirt eating, especially since it could stem from an unresolved psychiatric issue rather than a nutritional inadequacy. Until this is confirmed, medicinal therapies (options A, B, and C) would not be ideal.

**AI_E:** While our diagnosis for this child includes not only pica but also toxoplasmosis, these conditions seem to be separate concerns. Pica is characterized by the ingestion of non-food items, in this case, the boy's habit of eating dirt and sand. Despite this behavior, laboratory results for zinc, lead and iron are within the reference ranges, suggesting no underlying nutritional deficiencies that may cause pica. Toxoplasmosis, a disease caused by the protozoan Toxoplasma gondii, is likely due to the ingestion of parasites from the soil eaten as part of the pica behavior, thus these are interconnected problems. The assumption of this situation necessitates an intervention that will concurrently address both the boy's ingrained pica habit and manage the confirmed toxoplasmosis infection. Critical consideration should also be given to the social aspect of his behavior— the mother's similar past dirt-eating habit suggests a potential learned behavior. Given that health-related behaviors are often shaped by one's environment, exploring the context in which the child's pica has developed is essential. Taking this into consideration, clinical reasoning would lead us to think of strengthening the child's behavioral modifications, focusing not only on the child but possibly extending this intervention to the family setting as well. This implies the need for a professional who could offer counseling and behavioral strategies, guide the child, and assist the family. Applying this step-by-step clinical reasoning process and taking note of our initial assumptions, we may then conclude that a referral to a child psychiatrist (Option D) likely represents the most suitable management strategy. Psychiatrists are trained to conduct thorough psycho-social evaluations, provide necessary counseling, and work with families to ensure the adoption of healthier behaviors. Finally, answering the posed query, the most suitable next step in management for the 5-year-old boy with pica and confirmed toxoplasmosis would be to refer the patient to a child psychiatrist (Option D). This action would allow for a comprehensive approach to the situation addressing both the medical and behavioral concerns facing the child.

**AI_B:** RESTATING THE QUESTION: The question asks me to decide the best course of action for the management of a 5-year-old boy with confirmed toxoplasmosis and pica, the habit of eating nonnutritive substances like dirt, which he has had since the age of 3. The options are prescribing some medications, referring the patient to a dietician, or a child psychiatrist. Pica is an eating disorder that can lead to serious health problems like lead poisoning and iron-deficiency anemia. In this context, 'symmetrical nontender cervical adenopathy' refers to the swelling of the lymph nodes in the neck which are symmetric and are not painful upon touching. PLANNING: I am assuming that the child's eating disorder (pica) is due to some kind of micronutrient deficiency or behavioral issue. Given that blood test results show mineral levels are within the normal range, I should question this assumption to consider that the underlying cause of the pica could be more psychological or environmental rather than physiological. I hypothesize that he might be mimicking the mother's past behavior (the mother admitting that she used to eat dirt) and the possibility of neglect due to the mother's work schedule. CLINICAL REASONING: Considering this alternate assumption, the appropriate next step should address this psychological or environmental aspect. Behavioral and cognitive-based therapies delivered by a child psychiatrist can be effective in teaching the child to recognize and change unhelpful thinking patterns and behaviors. The psychiatrist would be able to discern whether the behavior is due to environmental influences or a more severe condition such as attention deficit hyperactivity disorder (ADHD) or pediatric autoimmune neuropsychiatric disorders associated with streptococcal infections (PANDAS) which can sometimes present with obsessive behaviors like pica. CONCLUDING: Given all of the above, referring the patient to a child psychiatrist seems to be a viable solution. This recommendation can potentially tackle both the psychological and environmental elements contributing to the pica behavior. RESPONSE: Thus, based on the clinical reasoning conducted, the answer would be (D) Refer the patient to a child psychiatrist. A child psychiatrist can provide a comprehensive evaluation and treatment plan taking into account both the environmental and psychological elements involved in this case, helping the child to manage his behavior.

**AI_D:** RESTATING THE QUESTION: The query is regarding a 5-year-old boy who has been eating dirt since he was 3 years old. He is currently asymptomatic, vital signs are normal and he is confirmed to be infected with Toxoplasma. The mother does confirm her own history of dirt-eating behavior which has now stopped. Laboratory tests including the evaluation of zinc, lead, and iron within his system return normal results. The question is asking for the most suitable next management step, given that the boy is already being prescribed pyrimethamine and sulfadiazine for the toxoplasmosis. The options include prescribing either fluoxetine, methylphenidate, risperidone, referring the patient to a child psychiatrist or to a dietician. PLANNING: Key points to note are that the child suffers from Geophagia which is the intentional ingestion of earth, soil, or clay. It's a form of pica, a medical disorder associated with the eating of nonnutritive substances for a period of at least one month, and inappropriate for the child's developmental level. Geophagia is noted in areas with poor nutrition and can lead to infections from fecal-oral transmissions like toxoplasmosis. In this case, it seems the practice comes from the learned behavior from the mother, as nutritional deficiencies have been ruled out. CLINICAL REASONING: Because the child's laboratory results are normal, it appears that his habit of dirt eating (pica) is not a result of any physiological deficiencies. Pica is often observed in children and can be due to a variety of factors, including nutritional deficiencies, psychological disorders, and learned behaviors. In this case, there is a potential element of learned behavior indicated by the mother's admission of her own past of dirt eating. CONCLUDING: Given that the mother has admitted to her own behavior, and the child does not demonstrate other behavioral abnormalities, it can be deduced that a behavioral management approach might be more effective. The child would certainly benefit from mental health intervention. There isn't enough information to suggest a dietary deficiency or malnutrition that would warrant a dietician's involvement, especially since serum tests return normal. RESPONSE: Therefore, the most appropriate next step in the management of this case would be to (D) Refer the patient to a child psychiatrist. This would underpin the need for therapeutic intervention and address any potential psychosocial issues contributing to the child's pica behavior.

**AI_C:** Restating the question, we are asked to determine the best course of action for a 5-year-old boy exhibiting pica behavior, which is defined as persistent eating of nonnutritive, nonfood substances. The boy's lab results, including his serum zinc, lead, and iron levels, are normal, ruling out any major mineral deficiencies that might explain his pica. Additionally, he has been diagnosed with toxoplasmosis, a disease that is commonly contracted from exposure to dirt and contaminants, possibly due to his pica behavior. The options given mostly involve prescribing certain medications or referring the patient to a specialist. Planning our response, the key assumption here is that pica, in this case, is a result of a behavioral, psychological, or perhaps an underlying psychiatric issue than a nutritional or physiological one. Given that the child's mother also admitted to a past habit of eating dirt, there could potentially be a behavioral or environmental component to this issue that could be better addressed through a psychiatric approach rather than a dietary one. Additionally, despite his pica, the child seems to be in normal health based on his lab results, height, weight, and BMI. This further supports the assumption that his pica is not a result of a physiological deficiency or disorder. Using clinical reasoning, we can connect the information given in the question to create a potential cause and effect scenario. The child's consumption of dirt has potentially increased his exposure to the parasite that causes toxoplasmosis, leading to his diagnosis. However, since there doesn't seem to be any anatomical, physiological, or nutritional cause behind his pica, it would be reasonable to infer that the issue may be behavioral or psychological. Concluding, it seems that among the given options, referring the child to a child psychiatrist could be the most effective course of action. A psychiatrist would be better equipped to deal with potential behavioral or psychological issues behind pica, and could work with the child (and possibly also the mother, given her past pica behavior) to develop strategies to mitigate this behavior. Therefore, I would suggest (D) Refer the patient to a child psychiatrist, as the most appropriate next step in management for this patient.

**Facilitator** The consensus statement synthesizes the responses of AI_A, AI_B, AI_C, AI_D, and AI_E as follows: The question at hand involves determining the most appropriate next step in managing a 5-year-old boy who exhibits pica, specifically geophagy (eating dirt), and has been diagnosed with toxoplasmosis. The boy's mother has a similar history, suggesting the behavior might be learned. Laboratory results, including serum zinc, lead, and iron concentrations, are within the reference ranges, indicating no nutritional deficiency. The key assumption is that the boy's pica is not due to a physiological deficiency but could be a result of a behavioral, psychological, or underlying psychiatric issue. This assumption is supported by the fact that the boy's mother also had a history of geophagy, suggesting a potential learned behavior. Additionally, the boy's mother's work schedule leaves him largely unsupervised, which could contribute to self-soothing behaviors like pica. Given these considerations, the most appropriate next step in management would be to refer the patient to a child psychiatrist. This approach would allow for a comprehensive evaluation of the boy's behavior, taking into account both environmental and psychological elements. A child psychiatrist could provide necessary counseling, work with the family to ensure the adoption of healthier behaviors, and develop strategies to mitigate the pica behavior. Therefore, the consensus response to the clinical query would be to (D) Refer the patient to a child psychiatrist. This response prioritizes the need for a more comprehensive understanding of the child's habitual dirt eating, especially since it could stem from an unresolved psychiatric issue rather than a nutritional inadequacy. Until this is confirmed, medicinal therapies (options A, B, and C) would not be ideal.
